# In Athletes, the Diurnal Variations in Maximum Oxygen Uptake Are More Than Twice as Large as the Day-to-Day Variations

**DOI:** 10.3389/fphys.2019.00219

**Published:** 2019-03-18

**Authors:** Raphael Knaier, Denis Infanger, Max Niemeyer, Christian Cajochen, Arno Schmidt-Trucksäss

**Affiliations:** ^1^Department of Sport, Exercise and Health, Faculty of Medicine, University of Basel, Basel, Switzerland; ^2^Department Medicine, Training and Health, Philipps-University Marburg, Marburg, Germany; ^3^Centre for Chronobiology, Psychiatric Hospital of the University of Basel, Basel, Switzerland; ^4^Transfaculty Research Platform Molecular and Cognitive Neurosciences, University of Basel, Basel, Switzerland

**Keywords:** circadian, chronotype, time of day, habitual, performance

## Abstract

In competitive sports any substantial individual differences in diurnal variations in maximal performance are highly relevant. Previous studies have exclusively focused on how the time of day affects performance and disregarded the maximal individual diurnal variation of performance. Thus, the aims of this study were (1) to investigate the maximum diurnal variation in maximum oxygen uptake (VO_2_max), (2) to compare the diurnal variation of VO_2_max during the day to the day-to-day variation in VO_2_max, and (3) to investigate if there is a time-of-day effect on VO_2_max. Ten male and seven female athletes (mean VO_2_max: 58.2 ± 6.9 ml/kg/min) performed six maximal cardiopulmonary exercise tests including a verification-phase at six different times of the day (i.e., diurnal variation) and a seventh test at the same time the sixth test took place (i.e., day-to-day variation). The test times were 7:00, 10:00, 13:00, 16:00, 19:00, and 21:00. The order of exercise tests was the same for all participants to ensure sufficient recovery but the time of day of the first exercise test was randomized. We used paired *t*-tests to compare the nadir and peak of diurnal variations, day-to-day variations and the difference between diurnal and day-to-day variations. The mean difference in VO_2_max was 5.0 ± 1.9 ml/kg/min (95% CI: 4.1, 6.0) for the diurnal variation and 2.0 ± 1.0 ml/kg/min (95% CI: 1.5, 2.5) for the day-to-day variation. The diurnal variation was significantly higher than the day-to-day variation with a mean difference of 3.0 ± 2.1 ml/kg/min (95% CI: 1.9, 4.1). The linear mixed effects model revealed no significant differences in VO_2_max for any pairwise comparison between the different times of the day (all *p* > 0.11). This absence of a time-of-day effect is explained by the fact that peak VO_2_max was achieved at different times of the day by different athletes. The diurnal variations have meaningful implications for competitive sports and need to be considered by athletes. However, the results are also relevant to research. To increase signal-to-noise-ratio in intervention studies it is necessary to conduct cardiopulmonary exercise testing at the same time of the day for pre- and post-intervention exercise tests.

## Introduction

In competitive sports any substantial individual differences in diurnal variations in maximal performance are highly relevant. As shown by several reviews (Chtourou and Souissi, 2012), the diurnal variation of short-duration performance has been examined considerably more extensively than that of long-duration endurance performance. Our systematic literature search (see Supplemental Methods) in Pubmed and Web of Science revealed 15 studies (Reilly and Baxter, 1983; Hill et al., 1988; Burgoon et al., 1992; Dalton et al., 1997; Deschenes et al., 1998; Reilly and Garrett, 1998; Atkinson et al., 2005; Brown et al., 2008; Chtourou et al., 2012; Souissi et al., 2012; Hill, 2014; Chin et al., 2015; Facer-Childs and Brandstaetter, 2015; Rae et al., 2015; Aloui et al., 2017), that have examined the effect of the time of day on endurance performance with inconclusive results (Table 1). In detail, five studies showed significant differences (Atkinson et al., 2005; Chtourou et al., 2012; Hill, 2014; Facer-Childs and Brandstaetter, 2015; Aloui et al., 2017), three studies showed differences in subgroups (i.e., different chronotypes) (Hill et al., 1988; Brown et al., 2008; Chin et al., 2015), and seven studies showed no significant differences (Reilly and Baxter, 1983; Burgoon et al., 1992; Dalton et al., 1997; Deschenes et al., 1998; Reilly and Garrett, 1998; Souissi et al., 2012; Rae et al., 2015) in maximum performance at different times of the day.

**Table 1:** Time of the day effects on maximum aerobic performance.

**References**	**Participants**		**Measurements**			**Assessed potential confounders**		**Results**	
	***N* (sex)**	**Training level**	**Time points (hh:mm)**	**Test**	**Outcome**	**Chronotype**	**Training time**	**Total**	**Variation (%)/significance^**a**^**
Aloui et al., 2017	11 (not reported)	Physical education students	07:00, 17:00	Shuttle-run test	Total distance (m)	Yes	Not reported	07:00: 891 ± 76 17:00: 1,149 ± 107	p < 0.05
Atkinson et al., 2005	8 (male)	Cyclists	07:30, 17:30	16.1 km bicycle ergometer time trial	Duration (s)	Yes	Not reported	07:30: 1,426 ± 104 17:30: 1,370 ± 99	p < 0.05
Brown et al., 2008	8 (male) 8 (female)	Trained rowers	05:00–07:00, 16:30–18:00	2000 m rowing ergometer time trial	Duration (s)	Yes	Not reported	Not reported	ECT: + 4.8 s faster in the morning (1.1%) ICT: n.s. / LCT: n.s.
Burgoon et al., 1992	26 (male)	Untrained	07:30–08:30, 19:30–20:30	Treadmill incremental test (Bruce protocol)	VO_2_max (ml/kg/min)	Yes	Not reported	07:30: 43.6 ± 5.6 19:30: 43.4 ± 4.8	n.s.
Chin et al., 2015	35 (male)	Athlete students	09:00–10:00, 12:00–13:00, 16:00–17:00	Shuttle-run test	Total distance (m)	Not reported	Not reported	Not reported	Not reported (only reported estimated VO_2_max differences)
Chtourou et al., 2012	20 (male)	Soccer players	07:00, 17:00	Shuttle-run test	Total distance (m)	Not reported	Not reported	07:00: 1,765 ± 485 17:00: 2,046 ± 535	p < 0.05
Dalton et al., 1997	7 (male)	Competitive cyclists	08:00–10:00, 14:00–16:00, 20:00–22:00	15 min bicycle ergometer time trial	Total work (kJ)	Not reported	Not reported	8:00: 278 ± 10 14:00: 277 ± 10 20:00: 277 ± 10	n.s.
Deschenes et al., 1998	10 (male)	Untrained	08:00, 12:00, 16:00, 20:00	Bicycle ergometer ramp test (30W/2min)	VO_2_max (ml/kg/min)	Not reported	Not reported	08:00: 52.0 ± 7.0 12:00: 55.1 ± 10.2 16:00: 56.7 ± 9.5 20:00: 56.9± 10.2	n.s.
Facer-Childs and Brandstaetter, 2015	20 (not reported)	Trained hockey players	07:00, 10:00, 13:00, 16:00, 19:00, 22:00	Shuttle-run test	Total distance (m)	Yes	Yes	Not reported	ECT: 7.6% ± 1.2 ICT: 10.0% ± 1.6 LCT: 26.2% ± 4.0
Hill et al., 1988	8 (male) 24 (female)	Not reported	06:00–08:30, 15:30–18:00	Bicycle ergometer incremental test (20W/min)	VO_2_max (l/min)	Yes	Not reported	ECT: 06:00: 2.75^b^ 15:30: 2.77^b^ LCT: 06:00: 2.64^b^ 15:30: 2.75^b^	n.s. < 0.05
Hill, 2014	20 (male)	Physically active	06:30–09:30, 17:00–20:00	Bicycle ergometer step test (50W/2min) constant-power test (100% of peak power)	VO_2_max (ml/kg/min) time to exhaustion (s)	Yes	Not reported	06:30: 52 ± 6 17:00: 54 ± 7 06:30: 275 ± 29 17:00: 329 ± 35	p < 0.05 p < 0.05
Rae et al., 2015	18 (male) 8 (female)	Trained swimmers	06:30, 18:30	200 m swimming time trial	Duration (s)	Yes	Yes	06:30: 159 ± 23 18:30: 159 ± 22	n.s.
Reilly and Baxter, 1983	8 (female)	Not reported	06:30, 22:00	Bicycle ergometer constant load test (95% of VO_2_max)	Time to exhaustion (s)	Not reported	Not reported	06:30: 260 ± 150 22:00: 436 ± 432	n.s.
Reilly and Garrett, 1998	7 (male)	Not reported	08:30, 17:30	Bicycle ergometer constant load test (70% of VO_2_max)	Time to exhaustion (min)	Not reported	Not reported	08:30: 17:30: 60.9 ± 6.6	n.s.
Souissi et al., 2012	12 (male)	Trained	14:00, 20:00	Shuttle-run test	Total distance (m)	Yes	Not reported	Not reported	n.s.
current study	10 (male) 7 (female)	Trained	07:00, 10:00, 13:00, 16:00, 19:00, 21:00	Bicycle ergometer incremental test (25W/min males) (20W/min females)	VO_2_max (ml/kg/min)	Yes	Yes	07:00: 55.4 ± 7.1 10:00: 55.8 ± 7.1 13:00: 55.0 ± 7.6 16:00: 54.3 ± 6.4 19:00: 56.8 ± 6.4 21:00: 55.6 ± 7.1	n.s.

a *None of the studies reported 95% confidence intervals for the differences*.

b *Standard deviation not reported*.

*ECT, early chronotype; ICT, intermediate chronotype; LCT, late chronotype; n.s., not significant; VO_2_max, maximum oxygen uptake*.

However, the main issue is that most studies measured performance only at two times of the day (Reilly and Baxter, 1983; Hill et al., 1988; Burgoon et al., 1992; Reilly and Garrett, 1998; Atkinson et al., 2005; Brown et al., 2008; Chtourou et al., 2012; Souissi et al., 2012; Hill, 2014; Rae et al., 2015; Aloui et al., 2017) with 07:00 and 17:00 being the most frequently used times. Such a large measurement interval carries a high risk of missing the peak and nadir of performance during the day. Consequently, these studies are unable to describe the full extent of diurnal variation in performance. Furthermore, all studies show several methodological shortcomings, including missing sample size calculations in all studies, sample sizes ≤ 12 in half of the studies (Reilly and Baxter, 1983; Dalton et al., 1997; Deschenes et al., 1998; Reilly and Garrett, 1998; Atkinson et al., 2005; Souissi et al., 2012; Aloui et al., 2017), not reporting the absolute data for the primary outcome (Brown et al., 2008; Souissi et al., 2012; Chin et al., 2015; Facer-Childs and Brandstaetter, 2015), and no information regarding the sequence of the investigated times of day or regarding a performed randomization. Additionally, some studies tested untrained or moderately trained participants (Hill et al., 1988; Burgoon et al., 1992; Deschenes et al., 1998), making the results not generalizable for athletes. Moreover, the majority of studies used more time efficient but also less precise methods such as shuttle-run tests. The measurement of maximum oxygen uptake (VO_2_max) in a cardiopulmonary exercise test (CPET), which is supposed to represent the gold standard to determine aerobic performance, was only measured in studies with a small sample size (Deschenes et al., 1998) or with large measurement intervals (Hill et al., 1988; Burgoon et al., 1992; Hill, 2014). Previous studies also do not consider exhaustion criteria, leaving room for speculation whether lower performance at a certain time of day is due to physiological (i.e., no higher performance possible) or psychophysiological (i.e., not motivated to perform with maximum effort) reasons.

In addition to the study design, data analysis in previous studies is debatable as these studies solely investigated the effects of daytime on maximum performance by comparing performances achieved at different times of the day on a group level. Because several factors such as habitual training time (Torii et al., 1992; Rae et al., 2015) or chronotype (Hill et al., 1988; Brown et al., 2008; Facer-Childs and Brandstaetter, 2015) seem to influence the time of day when peak performance is achieved, we expected it to be unlikely that all participants reach their peak and nadir of performance at the same time of day. If an athlete performs better in the morning and another athlete performs better in the afternoon, the differences would be canceled out on a group level leading to the false conclusion that there are no diurnal variations in maximum performance. Therefore, we additionally compared each participant's VO_2_max at the peak and nadir of the day to describe the diurnal variations in VO_2_max. Furthermore, we aimed to determine the day-to-day variation in athletes' VO_2_max to be able to put the possible diurnal variation into context.

The aims of this study, therefore, were to (1) investigate the maximum diurnal variation in VO_2_max, (2) to compare the diurnal variation of VO_2_max during the day to the day-to-day variation in VO_2_max, and (3) to investigate if there is a time of peak VO_2_max on a group level. A further aim was (4) to investigate to which extent chronotype and habitual training time contribute to the diurnal variation of VO_2_max during the day.

To address methodological shortcomings from previous studies, we used a measurement interval of 3 h (i.e., six times of the day) in our study, tested an adequate number of trained athletes in a randomized sequence using the gold standard for cardiopulmonary exercise testing, and considered exhaustion criteria.

## MaterialS and Methods

### Study Design

This study was conducted between December 2016 and May 2018 in the laboratory of the Department of Sport, Exercise and Health of the University of Basel, Switzerland. Participants gave written informed consent before inclusion, and the study was approved by the local ethics committee “Ethikkommission Nordwest- und Zentralschweiz” (EKNZ 2016-01572). To investigate the diurnal variation, participants performed CPET at 7:00, 10:00, 13:00, 16:00, 19:00, and 21:00. The order of CPET was the same for all participants to ensure sufficient recovery of at least 26 h. However, the time of day of the first CPET was randomized. To investigate the day-to-day variation, we performed a seventh CPET at the same time of day as the sixth CPET. 21:00 instead of 22:00 was chosen because this time represented the dimmed light melatonin onset in a study with athletes of comparable age (Knaier et al., 2017).

### Participants

Inclusion criteria were physical health, age between 18 and 40 years, no shift-work in the last 3 months, and no travel across time zones in the 4 weeks prior to the study. To avoid training effects resulting from performing multiple CPET and to be able to generalize the results for athletes, the inclusion criterion for VO_2_max was ≥ 50 ml/kg/min for males and ≥45 ml/kg/min for females achieved during the first CPET. This criterion was based on the 95th percentile of The American College of Sports Medicine reference values for VO_2_max (i.e., 56 ml/kg/min for males and 50 ml/kg/min for females). Because it may be possible that a participant performs his/her first CPET at the nadir of performance, we reduced the criterion for VO_2_max by 10% based on the expected maximum diurnal variation of VO_2_max during the day.

### Testing Procedure

The Munich Chronotype Questionnaire (Roenneberg et al., 2004) was used to determine individuals' midpoint of sleep on free days corrected for oversleeping due to sleep debt on workdays (MSFsc). Further, participants filled out a questionnaire about their habitual training times, and a questionnaire asking the athletes at which of the six times of the day they would expect to have the best VO_2_max. The same questionnaire was filled out again after the sixth CPET when participants had performed one CPET at each of the predefined times of the day. Subsequently, sex and age were recorded and body height and blood pressure were measured. Then, participants filled out the Physical Activity Readiness Questionnaire (Shephard, 1988) and underwent a physical examination by a physician that included 12-channel resting electrocardiography and assessment of medical history.

### Cardiopulmonary Exercise Test

Immediately before each CPET, body mass (kg) and body fat mass (kg) were measured with four-segment bioelectrical impedance analyses (Inbody 720, Biospace, Seoul, South Korea). Subjective sleepiness was assessed before CPET with the Karolinska Sleepiness Scale (Kaida et al., 2006) ranging from 1 (“extremely alert”) to 9 (“very sleepy, great effort to keep alert”). CPET was performed on a bicycle ergometer (Sport Excalibur, Lode Medical Technology, Groningen, The Netherlands) under standardized laboratory conditions (air humidity 40–55%, room temperature 20–22°C). For male/female participants the exercise protocol consisted of a warm-up of 5 min at 75/50 W, a linear increase of workload with 25/20 W/min up to exhaustion, and a 10 min cool-down phase at 75/50 W. Throughout the entire CPET, participants were verbally encouraged by the test supervisors to perform with maximum effort in all tests. Gas exchange was measured breath-by-breath (MetaMax 3B, Cortex Biophysik GmbH, Leipzig, Germany) with the highest 30 consecutive seconds of VO_2_ being determined as VO_2_max. After the cool-down phase a VO_2_max verification test was performed. Therefore, workload was set to 50% of maximum power output achieved during CPET for 2 min, then increased to 70% for 1 min and in the final stage of the verification test to 105% of maximum power output until exhaustion. VO_2_max verification was accepted if the verification-VO_2_ was ± 3% of the initially measured VO_2_max (Nolan et al., 2014). Because VO_2_-plateau was not reached in all tests and VO_2_-verification data from this study showed low agreement for diurnal variations (data not shown—under review), secondary exhaustion criteria were used to verify VO_2_max. During the first CPET, heart rate was measured with 12-channel electrocardiography and in the subsequent CPETs via 3-channel electrocardiography (Custo med GmbH, Ottobrunn, Germany). In all tests, heart rate was additionally measured with a heart rate belt (Polar T-34, Polar Electro Europe AG, Zug, Switzerland). Maximum heart rate was determined by the values recorded with the heart rate belt. Ratings of perceived exertion was assessed according to the 6–20 Borg scale (Borg, 1982). Blood lactate concentration was measured at rest, immediately after exhaustion, and at minutes one, three, and five of the cool-down phase. Blood samples were analyzed immediately after the exercise test (SuperGL Ambulance, Hitado Diagnostic Systems, Moehnesee, Germany). All participants reached all of the following exhaustion criteria in every test: respiratory exchange ratio ≥1.1, heart rate ≥95% of predicted maximum heart rate [210—age (years)], ratings of perceived exertion ≥19, and blood lactate concentration ≥8 mmol/l. These cut-off values were based on a previous study with athletes (Knaier et al., 2017) and proofed to strongly reduce type I errors without increasing the chance of type II errors (Knaier et al., 2018).

### Observational Phase

Participants were advised to restrain from alcohol and sport during the entire study duration and to keep a constant sleeping routine. To monitor the compliance, participants filled out a diary recording bedtime, sleep time, wake-up time, and subjective sleep quality. Compliance to restrain from sport was monitored objectively by wGT3X+ ActiGraphs (Pensacola, United States) throughout the entire study.

### Statistical Analysis

The primary outcome of this study was the difference in VO_2_max (in ml/kg/min) between the CPET with the lowest (i.e., nadir) and the CPET with the highest (i.e., peak) VO_2_max (i.e., maximum diurnal variation). We used paired *t*-tests to compare the nadir and peak of diurnal variations, day-to-day variations and the difference between diurnal and day-to-day variations. To calculate the day-to-day variations we compared the highest and lowest value from the sixth and the seventh test irrespective of which of these values was recorded in the first or second test. To investigate if there is a time of peak VO_2_max on a group level, we used descriptive statistics (i.e., boxplots) and a linear mixed effects model with *post-hoc* tests to compare the VO_2_max achieved at different times of the day. Habitual training time was coded as four different dummy variables, which were set to “1” if a participant trained during one of the predefined timeframes on any day during a usual week and to “0” if not. The timeframes were (Table 2): 04:00–08:59 (morning), 09:00–13:59 (noon), 14:00–18:59 (afternoon/evening), and 19:00–23:59 (evening/night). We used proportional odds logistic regression models to model the probability of achieving the peak VO_2_max in a certain time frame (Hosmer et al., 2013). The midpoint of sleep (MSFcs) and the dummy variables for the habitual training times were included as predictors. Likelihood ratio tests were used to assess statistical significance. Furthermore, we used descriptive statistics (i.e., histograms) to show the percentage of participants reaching their peak VO_2_max earlier during the day (i.e., at 16:00 and earlier), and reaching it later during the day (i.e., at 19:00 or later) for the two groups “morning types” and “evening types.” Median split of MSFsc was used to allocate participants to the groups. Furthermore, we performed a sensitivity analysis to investigate if there were learning (i.e., increase in performance) or fatigue effects (i.e., decrease in performance) from the first to the last CPET using a linear mixed effects model with test times in chronological order as continuous predictors. For each analysis, we report the estimated differences with 95% confidence intervals (95% CI) in outcome between the groups. For our analyses and graphics, we used IBM SPSS Statistics for Windows, version 24 (IBM Corp., Armonk, N.Y., USA) and R version 3.5.0 (R Foundation for Statistical Computing, Vienna, Austria), respectively.

**Table 2:** Participant characteristics—median (interquartile range).

**Characteristic**	**Males (*n* = 10)**	**Females (*n* = 7)**
Age (years)	26 (23; 33)	27 (23; 34)
Height (cm)	179 (173; 184)	168 (166; 174)
Body mass (kg)	73 (68; 82)	64.9 (60.6; 68.6)
Body fat content (%)	12 (10; 14)	22 (17; 28)
**CPET**		
Pmax (W)	375 (352; 408)	293 (261; 318)
VO_2_max L/min	4.38 (4.23; 4.77)	3.29 (3.11; 2.75)
VO_2_max mL/kg/min	61.5 (57.5; 67.5)	54.3 (46.6; 56.7)
Sleepiness on KSS	2.5 (1.8; 3.3)	3.0 (2.0; 5.0)
**CHRONOTYPE**		
MSFsc (h)	3.61 (3.11; 5.07)	3.55 (2.52; 3.77)
**PARTICIPANTS' TRAINING HABITS [% TRAINED BETWEEN …]**^***a***^		
04:00–08:59	30%	14%
09:00–13:59	90%	71%
14:00–18:59	70%	71%
19:00–23:59	80%	71%

a *Multiple answers possible*.

*CPET, cardiopulmonary exercise test; Pmax, maximum power output; VO_2_max, maximum oxygen uptake; KSS, Karolinska Sleepiness Scale (range: 1 [extremely alert] to 9 [very sleepy]); MSFsc midpoint of sleep on free days corrected for oversleep due to sleep debt on workdays (MSFsc)*.

### Sample Size Calculation

For the sample size calculation, we assumed that the VO_2_max achieved at the time of peak VO_2_max was 60 ± 6 ml/kg/min (Knaier et al., 2017). Based on the previous reported difference of 10% in intermediate chronotypes and of 11.6% in all participants, we expected the VO_2_max achieved at the time of nadir VO_2_max to be 54 ± 6 ml/kg/min. Furthermore, we expected to reduce error variability by conservatively assuming a correlation of 0.5 between the VO_2_max from the peak and VO_2_max from the nadir of the day. With a 2-sided significance level of 0.05, the sample size needed to attain a targeted power of 90% to show a significant difference between the peak and nadir of VO_2_max was 13 participants. We increased this sample size to 18 participants to ensure an equal number of three participants in each of the six groups (i.e., the six different starting times for the first CPET). For our sample size calculation, we used G^*^Power (University of Kiel, Kiel, Germany).

### Randomization

We used Friedman's Urn Model [UD(1, 0, 2)] to allocate the participants to the different starting times (Wei, 1978; Smith, 2014). This randomization procedure is known to lead to more balanced groups if the number of groups is large compared to the number of participants while still being free of selection or accidental bias (Wei, 1978). Participant recruitment, therefore, continued until three participants were allocated to each of the six starting times for CPET.

## Results

### Participant Flow and Characteristics

**Figure 1:**
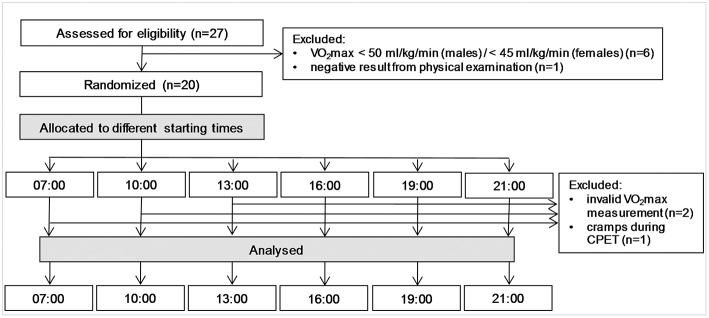
Flow of participants through the trial.

Twenty-seven participants were assessed for eligibility, whereby six participants did not meet the inclusion criteria for VO_2_max. One participant was excluded due to medical reasons. The remaining 20 participants were randomly allocated to the six different starting times (Figure 1). Two participants were excluded due to technical problems of measuring VO_2_ and one participant due to medical reasons. Participants' characteristics from the CPET with the highest VO_2_max are presented in Table 2. None of the participants was an exceptionally early or late chorotype. Participants followed the instructions regarding sleeping routine and physical activity.

### Diurnal and Day-to-Day Variation in VO_2_max

Mean and standard deviation of VO_2_max in ml/kg/min, VO_2_max in L/min and Pmax at the time of the peak and the time of the nadir are presented in Table 3. There were significant diurnal variations for all three parameters as indicated by the mean differences. Furthermore, for all parameters there were significant correlations between the values from the peak and the nadir of the day. These correlations were higher than the conservatively assumed 0.5 from the sample size calculation.

**Table 3:** Diurnal variation and day-to-day variation for different performance parameters.

**Characteristic**	**Peak (*n* = 17)**	**Nadir (*n* = 17)**	**Mean difference ± SD (95% CI)**	**Pearson's correlation r (*p*)**
**DIURNAL VARIATION**				
VO_2_max (mL/kg/min)	58.2 ± 6.9	53.2 ± 6.5	5.0 ± 2.0 (4.1, 6.0)	0.961 (*p* ≤ 0.001)
VO_2_max (L/min)	4.06 ± 0.72	3.72 ± 0.71	0.34 ± 0.14 (0.27, 0.41)	0.989 (*p* ≤ 0.001)
Pmax (W)	348.7 ± 61.3	328.7 ± 58.1	20.0 ± 9.4 (15.2, 24.8)	0.981 (*p* ≤ 0.001)
**DAY-TO-DAY VARIATION**				
VO_2_max mL/kg/min	56.7 ± 7.1	54.7 ± 7.0	2.0 ± 1.0 (1.5, 2.5)	
VO_2_max L/min	3.94 ± 0.75	3.81 ± 0.74	0.13 ± 0.09 (0.08, 0.17)	
Pmax (W)	343.5 ± 61.7	338.6 ± 61.5	4.9 ± 5.3 (2.2, 7.6)	

*Pmax, maximum power output; VO_2_max, maximum oxygen uptake; SD, standard deviation; 95% CI; 95% confidence interval*.

There were also significant day-to-day variations for all three performance related parameters (Table 3).

**Figure 2:**
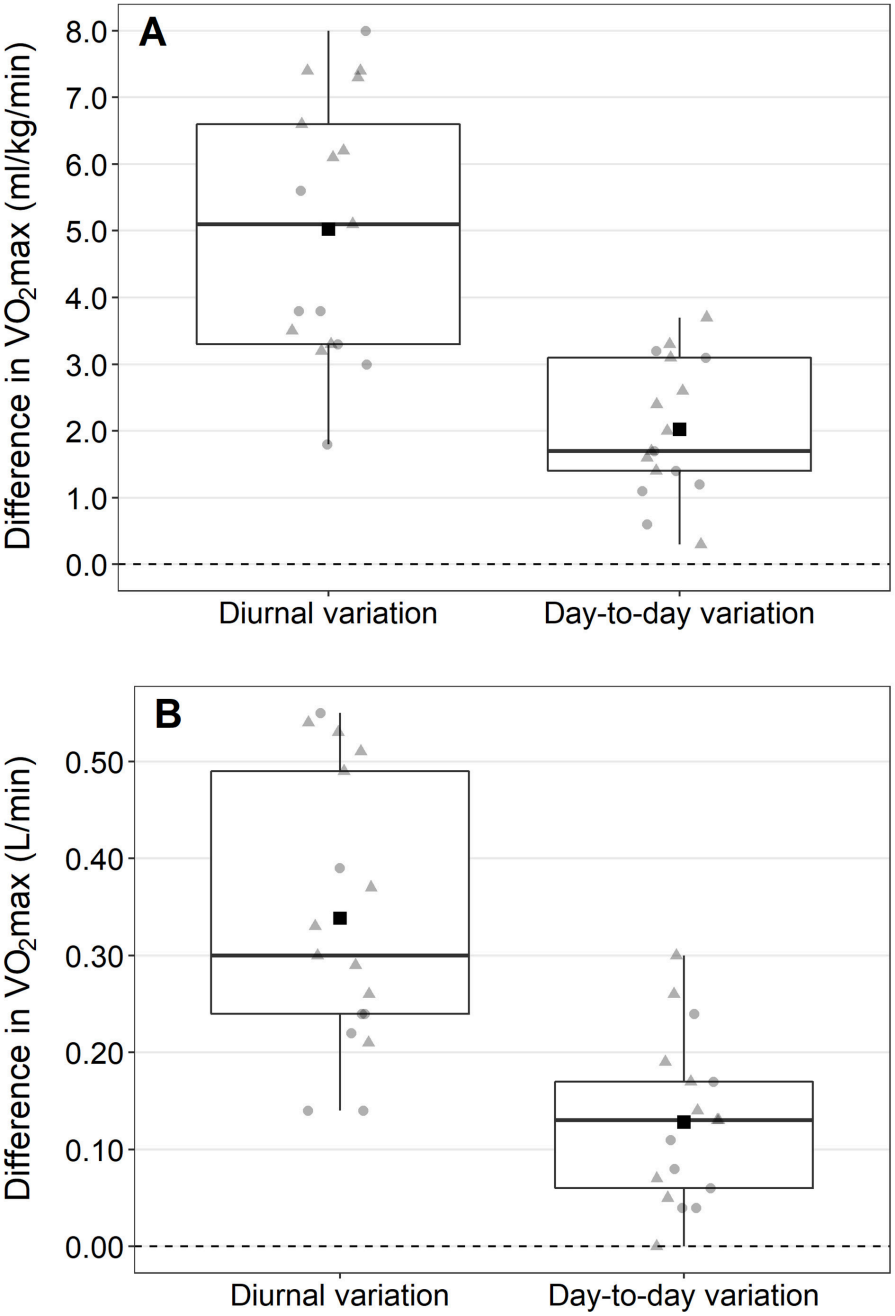
Differences between the cardiopulmonary exercise test with the highest and the exercise test with the lowest maximum oxygen uptake (VO_2_max) during the day (i.e., diurnal variation) and between the two exercise test taking place at the same time of the day (i.e., day-to-day variation). Values are presented for VO_2_max in ml/kg/min **(A)** and l/min **(B)**. Square, mean difference; triangles, males; circles, females.

For all three performance parameters the diurnal variations were significantly higher than the day-to-day variations with a mean difference of 3.0 ± 2.1 ml/kg/min (95% CI: 1.9, 4.1), 0.21 ± 0.17 L/min (95% CI: 0.12, 0.30), and 15.1 ± 11.8 W (95% CI: 9.0, 21.1), respectively. The diurnal variations were 2.5 to 4 times greater than the day-to-day variations for all three parameters. Figure 2 shows the contrast in diurnal and day-to-day variations in VO_2_max in ml/kg/min. Pearson's correlation showed no relationship between the diurnal variation in VO_2_max and the day-to-day variation in VO_2_max between the participants (*r* = 0.050, *p* = 0.848).

### Influence of Time of Day on VO_2_max

**Figure 3:**
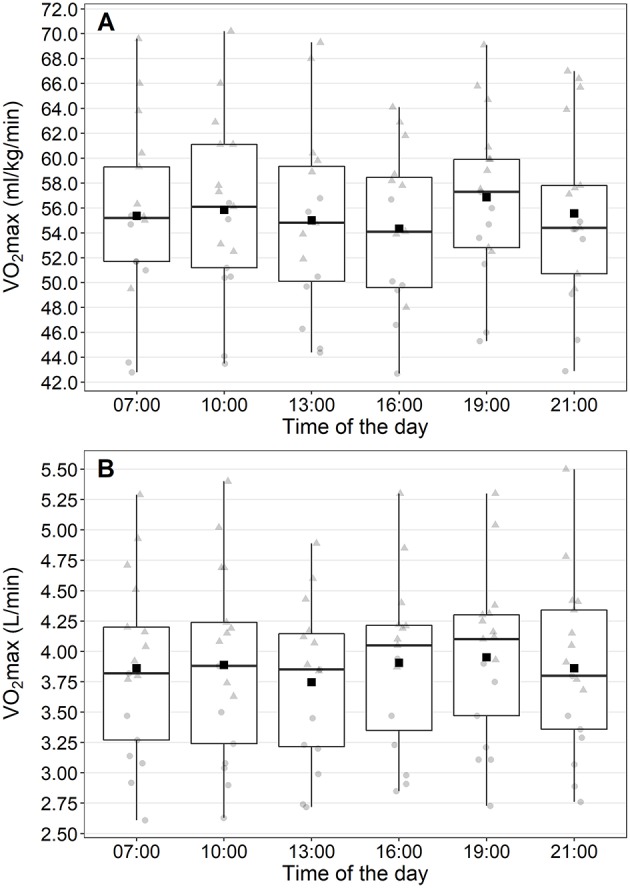
Maximum oxygen uptake (VO_2_max) at the different times of the day. Values are presented for VO_2_max in ml/kg/min **(A)** and l/min **(B)**. Square, mean values; triangles, males; circles, females.

**Figure 4:**
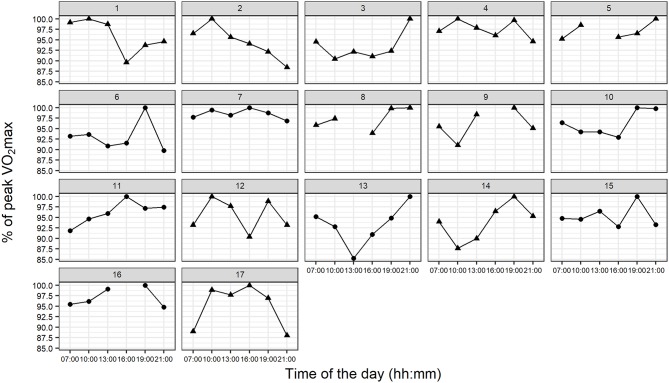
Individual profiles for the diurnal variation in percentage of peak VO_2_max from all participants. Triangles, males; circles, females.

The linear mixed effects model revealed no significant differences in VO_2_max between the different times of day (Figure 3). Neither the fixed effect of time ($\chi_{5}^{2}$ = 8.00, *p* = 0.156) nor any pairwise comparison between the time points reached statistical significance (all *p* > 0.11). Time of day when peak VO_2_max was achieved was nearly equally distributed between the times 10:00 (*n* = 4), 16:00 (*n* = 3), 19:00 (*n* = 5), and 21:00 (*n* = 4). Interestingly, no participant achieved his/her peak VO_2_max at 13:00. The individual profiles for the diurnal variation differed between participants (Figure 4).

### Influence of Chronotype and Habitual Training Time on the Time of Peak VO_2_max

**Figure 5:**
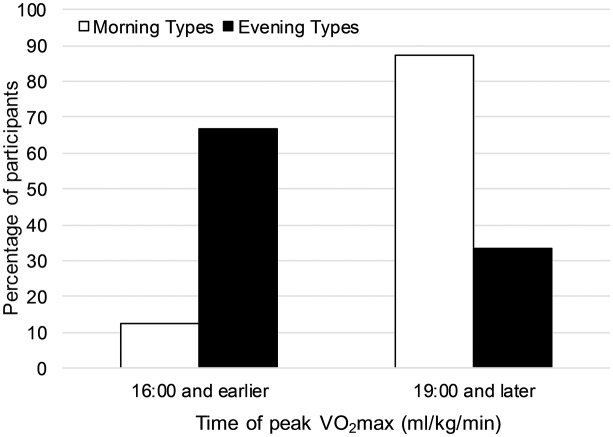
Percentage of participants reaching their peak VO_2_max (ml/kg/min) earlier during the day (i.e., at 16:00 and earlier) and reaching it later during the day (i.e., at 19:00 or later) for the two groups “morning types” and “evening types”.

There was little evidence (*p* = 0.129) that the habitual training time influenced the time of day at which participants reached their peak VO_2_max since no effect reached statistical significance. Chronotype had a significant influence on the time of day when peak VO_2_max was achieved (OR = 0.34, 95%-CI: 0.11, 0.88, *p* = 0.035). Participants with later MSFsc had a higher probability to reach their peak VO_2_max earlier during the day than participants with earlier MSFsc. Figure 5 shows the percentage of participants reaching their peak VO_2_max earlier during the day (i.e., at 16:00 and earlier) and reaching it later during the day (i.e., at 19:00 or later) for the two groups “morning types” and “evening types.”

### Training Effects, Participants' Expectation, Adverse Events, and Exhaustion Criteria

The trial when peak VO_2_max (ml/kg/min) was achieved was nearly equally distributed between the trials: T1 (*n* = 1), T2 (*n* = 2), T3 (*n* = 4), T4 (*n* = 3), T5 (*n* = 3), T6 (*n* = 4). The linear regression analysis showed little evidence for training effects from the first to the last CPET ($\chi_{1}^{2}=$ 1.80, *p* = 0.179). None of the participants expected to reach their peak VO_2_max at 07:00, which proved to be true. Seven out of 17 participants anticipated their time of peak VO_2_max correctly. Except for one participant that was excluded because of cramps during the CPET, no further severe adverse events occurred during the study. The median (IQR) for the secondary exhaustion criteria at the time of peak VO_2_max (ml/kg/min) were maximum respiratory exchange ratio 1.23 (1.18, 1.26), maximum heart rate 187 (183, 197) and maximum rating of perceived exertion 20 (20, 20), respectively. At the time of the nadir of VO_2_max the values were maximum respiratory exchange ratio 1.23 (1.16, 1.26), maximum heart rate 186 (180, 192) and maximum rating of perceived exertion 20 (20, 20), respectively.

## Discussion

This is the first study showing that diurnal variations in VO_2_max can be present without time-of-day effects. In line with several previous studies (Reilly and Baxter, 1983; Hill et al., 1988; Burgoon et al., 1992; Dalton et al., 1997; Deschenes et al., 1998; Reilly and Garrett, 1998; Brown et al., 2008; Souissi et al., 2012; Rae et al., 2015), we found no significant effect of the time of day on peak aerobic power. However, this was not due to the absence of diurnal variations, but rather to the fact that peak VO_2_max is achieved at different times of the day by different athletes and therefore masks the time-of-day effect. Furthermore, we were able to show that the presented diurnal variation in VO_2_max of 5.0 ± 1.9 ml/kg/min is 2.5 times greater than the day-to-day variation in VO_2_max of 2.0 ± 1.0 ml/kg/min, which is highly likely to have a striking impact on athletes. Similarly, for VO_2_max in L/min and Pmax the diurnal variations were 2.5 and 4 times greater than the day-to-day variations. Interestingly, only three participants (18%) showed their nadir of VO_2_max at 07:00—the time of day of the fewest competitions. Thus, the reported relevant diurnal variation is present at times when most competitions take place.

Previous studies reported an influence of habitual training time on the time of day when peak performance is achieved (Rae et al., 2015) and that training at a specific time of day can reduce diurnal variations (Torii et al., 1992). However, we chose a realistic free-living setting with trained athletes and found that most endurance athletes train at several times during the day to balance their training load with other obligations. Because athletes already train at different times of the day, this, therefore, may reduce the relevance of training habits on the time of peak VO_2_max and diurnal variations.

In contrast to Facer-Childs and Brandstaetter (2015), we did not find the association of early chronotypes reaching peak performance earlier during the day and late chronotypes reaching it later during the day. In fact, we found the opposite. Participants with later MSFsc had a higher probability to achieve peak VO_2_max at 10:00 or 16:00 than 19:00 or 21:00 (Figure 5). Several methodological differences between the two studies may explain the inconclusive results. Facer-Childs and Brandstaetter (2015) did not use MSFsc as a continuous measure for chronotype, but grouped athletes into the three categories early, intermediate, and late chronotype. This grouping resulted in an inadequate sample size of five participants in both the early and the late group. Furthermore, in Facer-Childs and Brandstaetter (2015), participants had to perform only five tests out of the six tests taking place at the different times of the day and the sequence of test times was not reported to be randomized. Finally, the authors did not explain the high differences in time of day when peak performance was reached between the three chronotype groups. Early and intermediate chronotypes were reported to reach peak performance 5.6 and 6.5 h after an entrained wake-up time, respectively, while late chronotypes needed 11.2 h to reach peak performance.

We are aware that this study was not powered to show the association between chronotype and diurnal variation. However, the results from our well-controlled study suggest that the association between time of peak VO_2_max and habitual training time or chronotype seems to be more complicated and not as simple and linear as reported by Facer-Childs and Brandstaetter (2015). Similarly, it is most likely that physiological factors, which are supposed to explain the time of day effects in physical performance, are also not simple and linear. Changes in core body temperature is the most frequently used explanation for the peak of performance in the late afternoon and early evening. However, Atkinson et al. (2005) demonstrated more than a decade ago that body temperature before an exercise test cannot solely explain the time of day differences in performance. In detail, athletes still performed better in a time trial at 17:30 following a short warm-up than at 07:30 even after a vigorous warm-up for 25 min, although post warm-up temperatures did not differ between the two times of the day. Our study was planned as a proof of concept to show actual diurnal variations independent of time of day effects, and we, therefore, refrained to measure any physiological parameters such as body temperature or melatonin. However, to explain the underlying physiological mechanism of diurnal variation in performance, further studies are necessary. These studies should include the measurement of a broad range of possible and expected confounders such as body temperature, melatonin, testosterone, chronotype, training time, and sleep times. The knowledge about the interaction of these factors with the time of peak VO_2_max might help to predict the time of peak performance and shift it to the time of competition. Furthermore, we observed high differences in VO_2_max between two neighboring measurement points in some athletes indicating a rapid increase or decrease in performance. These athletes seem to be most vulnerable to changing competition times. In a previous study, evening bright light exposure showed a trend to increase maximum cycling performance through reduced melatonin levels (Knaier et al., 2017). This method might be most beneficial for athletes with rapid decreases in performance in the evening.

## Conclusions

Athletes show significant diurnal variations in VO_2_max, which are more than twice as large as the day-to-day variations. In competitive sports, these diurnal variations are highly relevant, because there are substantial differences in the time of the day when individuals achieve their peak performance. However, the direct application of our results for competitive sports is limited. We were able to show that some athletes have clear disadvantages if their time of peak performance does not comply with the time of competition, but further studies are required to investigate the underlying physiological mechanism causing these diurnal variations and to demonstrate methods to shift an athlete's time of peak performance. However, the results are directly applicable to exercise testing in research, athletes, and in a clinical setting. We could clearly show that it is necessary to conduct cardiopulmonary exercise testing at the same time of the day for pre- and post-intervention exercise tests to increase signal-to-noise-ratio. VO_2_max was not significantly higher at a specific time of the day compared to other times of the day. This absence of a time-of-day effect is explained by the fact that peak VO_2_max was achieved at different times of the day by different athletes. Habitual training times seem to have no influence on the time of day when peak VO_2_max is achieved, whereas the participants' chronotype may have an impact.

## Data Availability

The datasets generated for this study are available on request to the corresponding author.

## Author Contributions

RK, CC, AS-T: concept and design; RK: data acquisition; RK, DI, and MN: data analysis and interpretation; DI: statistical expertise; RK: writing manuscript; DI, MN, CC, and AS-T: writing—review and editing.

### Conflict of Interest Statement

The authors declare that the research was conducted in the absence of any commercial or financial relationships that could be construed as a potential conflict of interest.

## Abbreviations

VO_2_max: maximum oxygen uptake
CPET: cardiopulmonary exercise test
MSFsc: midpoint of sleep on free days corrected for oversleeping due to sleep debt on workdays.
