# Supplementary material for: In Athletes, the Diurnal Variations in Maximum Oxygen Uptake Are More Than Twice as Large as the Day-to-Day Variations
Source: Front Physiol. 2019 Mar 18;10:219. doi: 10.3389/fphys.2019.00219 (PMC6431663; doi:10.3389/fphys.2019.00219)
Supplement: Supplementary file 1 [file Data_Sheet_1.PDF]

SUPPLEMENTAL METHODS

Systematic literature review:

To the best of the authors' knowledge, there is no systematic review regarding the effects of the time of the day on physical performance that is up to date or was performed according to PRISMA guidelines. Recently published reviews (Souissi et al., 2012; Thun et al., 2015) did not perform systematic literature review and therefore missed several relevant studies. We performed a search in PubMed and Web of Science with the following two search strings:

PubMed: ((((effect OR dependence OR influence OR impact) AND ((time AND day) OR circadian rhythm [Mesh]) AND (athletic performance [Mesh] OR oxygen consumption [Mesh] OR muscle strength [Mesh] OR Exercise Test [Mesh] OR power output OR 1-RM OR VO2max OR VO2peak)))

Web of Science: (((((effect OR dependence OR influence OR impact) AND (diurnal OR circadian) AND (athletic performance OR muscle strength OR Exercise Test OR power output OR 1-RM OR VO2max OR VO2peak OR step test OR treadmill test OR ergometry test OR physical fitness test OR cardiorespiratory fitness OR physical endurance OR sports performance))))).

We found 2230 studies in PubMed and 859 studies in Web of Science, respectively. From the 3089 studies, 150 were duplicates, 2699 were excluded after screening the titles and further 171 studies after screening the abstracts. Furthermore, we excluded congress abstracts (n=1), reviews (n=9), studies without available full text (n=5). From the remaining 54 studies, only nine studies investigated time of the day effects on maximum performance in long-duration performance. From the nine studies found by our search strings, six were not mentioned in previous reviews.

In a second step we reviewed the literature list from two previously published reviews (Souissi et al., 2012; Thun et al., 2015) and found another four studies that matched our inclusion criteria. By reviewing the reference lists of the so far included 13 studies we found another two studies that were included in our overview (Table 1).

Souissi, M., Chtourou, H., Zrane, A., Cheikh, R. B., Dogui, M., Tabka, Z., et al. (2012). Effect of time-of-day of aerobic maximal exercise on the sleep quality of trained subjects. *Biol. Rhythm Res.* 43, 323–330. doi:10.1080/09291016.2011.589159.

Thun, E., Bjorvatn, B., Flo, E., Harris, A., and Pallesen, S. (2015). Sleep, circadian rhythms, and athletic performance. *Sleep Med. Rev.* 23, 1–9. doi:10.1016/j.smrv.2014.11.003.
